# Gynecological and Obstetrical Society of Baltimore, Md.

**Published:** 1891-02

**Authors:** William S. Gardner

**Affiliations:** Secretary


### Society Reports.

### GYNECOLOGICAL AND OBSTETRICAL SOCIETY OF BALTIMORE, MD.

### DECEMBER MEETING.

Vice-President Dr. Charles H. Riley in the chair.

Dr. Wm. E. Moseley related the following case :

Mrs. Maggie G., a light colored woman, about thirty years of age, twice married, has had two children by her first husband. Had suffered much during the past twelve years from dysmenorrhœa ; had been unable to do ordinary work.

Examination showed the uterus to be retroflexed and firmly bound down ; but the character of the adhesions could not be definitely made out. There was an irregular shaped elastic mass in the position of either tube diagnosticated as cystic ovaries, together with chronically inflamed tubes. All the pelvic tissues, were very sensitive to pressure. There was a deep, double laceration of the cervix, and a lacerated perineum, with very lax vaginal wall but only slight rectocele.

When the abdomen was opened the mass on either side of the pelvis was found to be composed of a cystic ovary and the corresponding tube firmly matted together by old organized adhesions, each mass being firmly bound down to the pelvic wall by numerous strong and many more recent adhesions. There were also adhesions to the omentum. The left ovary ruptured before it could be removed. The mass in the right side appeared to be a large hematosalpinx, but examination proved it to be an ovarian cyst into which blood had entered from a ruptured graafian follicle. The adhesions behind the uterus were very broad, strong bands, and were pulled off the uterine walls. All possible care was used to secure the patient against hemorrhage and the, abdomen was douched out with hot boiled water until the return flow was practically colorless. A glass perforated drainage tube was introduced to the bottom of the cul-de-sac, and the incision closed about it. The extreme difficulty of separating the adhesions and the douching prolonged the operation to about one and a half hours.

Although stimulants and artificial heat were pushed no reaction could be obtained, the temperature never reaching 95 degrees, and the patient died about six hours after the operation, apparently from shock. At no time was there any discharge of blood or even bloody fluid from the drainage tube. Dr. N. G. Keirle, however, kindly examined the pelvic cavity *post mortem*, and reported that death was due to hemorrhage, the exact source of which could not be made out.

Dr. J. Whitridge Williams kindly furnished the pathological report which will be given below.

Dr. Thomas Opie exhibited a placenta that he had gotten a few hours before the meeting from a case of placenta prævia.

The patient was thirty-five years of age and had borne one child previously. When he saw her first she was blanched and exsanguined. The blood flow begun three days before with a loss of a quart, and continued with more or less rapidity up to the time of operation. Her confinement was not expected for two weeks. When first seen by him there were some rythmical pains and some dilatation. The cervix was dilated with the fingers and cone of the hand ; the placenta was detached with a sweep of the forefinger around the cervix; the bag of waters was artifically ruptured and traction-rod forceps applied. The child was delivered in fifteen minutes without further loss of blood, the placenta coming away simultaneously with the birth of the child. Though the position was occiput posterior, there was no laceration of the perineum and the child was unscathed. Both mother and child were left doing well.

Dr. Opie also exhibited an ovarian tumor which he had recently removed. The tumor had developed into the epigastric region, and the abdomen was about as large as it would have been at the full term of pregnancy. It took two hours to break up the adhesions, which were very dense between the tumor and the intestines, and between the tumor and the omentum. The second tumor was taken from the pelvis. It was ovoidal in form, about seven inches in length, by five inches high and four inches thick. It was removed entire, and upon section it proved to be a dermoid growth. There was no history of peritonitis to account for the extensive adhesions. The patient had never had a day's discomfort, other than from the size of the cyst. She did not know until four months ago that she had a tumor. The material in the large cyst was colloid. Notwithstanding the extensive adhesions, the length of time consumed in breaking them up, and the injury resulting from the operation, the patient has made a good recovery, this being the seventeenth day after the operation.

Dr. Howard A. King: The term colloid is often used in two senses. An incorrect one, describing the yellowish, more or less opalescent, thick, viscid material, often found in ovarian cysts; it is employed in such cases as more or less synonymous with gluey. The other use of the term is to describe a rare condition, in which the contents of the cyst are more like calf's-foot jelly, and have a vitreous fracture; they are with great difficulty removed, clinging to everything. This latter is true colloid, and when found such tumors are of a suspiciously malignant character. We should limit the use of the word to the latter condition.

I wish to refer to two minor matters of interest suggested by this specimen of placenta prævia. The position which the placenta has occupied in the uterus can accurately be determined by the position of the opening in the membranes made by the passage of the child, inasmuch as the fundus uteri must of necessity be just opposite to this perforation. We can, therefore, by reconstructing the membranes see just in what part of the uterus the placenta lay. In one of my placenta prævia cases there was no hole at all in the membranes, as I had extracted the dead child through a perforation in the placenta. We can do still more than this in the way of a diagnosis with the membranes. By allowing them to be expelled untouched into the bed and carefully observing their exact position, we can tell as well on which side of the uterus the placenta was attached.

The second point is that we may have placenta prævia hemorrhage without being able to detect a placenta margin, owing to a low attachment of part of the placenta, near the internal os, below the contraction ring, but not over the hole of the cervical canal. The lower part of a placenta thus attached is separated by the opening up of the lower uterine segment.

Dr. L. E. Neale said: Although Dr. King had alluded to a point of some interest, it is of far more practical importance to recognize placenta prævia prior to its expulsion, and as far as he knew this could only be done with certainty by digital examination; partial placental separation and rupture of the membranes during labor in cases of placenta prævia was outlined by Mariceau as early as 1668, but was fully described by Puzas in 1759; he saw nothing in the history of the present case, as related by Dr. Opie, that contraindicated the method of Broxton Hicks, a method that up to the present time had given by far the best results, viz., 4½ per cent. maternal mortality. If this method when practicable could be performed earlier than delivery by any other method, and was not difficult and gave the best results, why not have applied it in the present case?

Dr. Wilmer Brinton asked why Dr. Opie objected to the tampon in cases of placenta prævia; he thought no arbitrary law could be applied.

Dr. Opie said, in closing the discussion, that results of operative procedure depended largely upon the skill and familiarity of the operator with the special operation resorted to; in his first case of placenta prævia he had attended to he had turned and lost both mother and child; with rapid dilatation and the forceps he feels that he has command of the situation, and having resorted to that method repeatedly, has gained greater skill and does better work. While Dr. Neale might do better by some other method, he is fully satisfied that he does best himself with the forceps; he is opposed to the use of the tampon because it conceals what is going on; it is not best to wait for pains; he is in favor of rapid dilatation and delivery in placenta prævia, in puerperal eclampsia and in abortion; to put in a tampon and go away is hazardous; the tampon is of very little help in hemorrhages.

Dr. Howard A. Kelly read a paper upon the palpation of the "Normal Uterine Appendages," (published in full in the February number of the *American Journal of Obstetrics.*) He stated that the normal uterine appendages could always be palpated. There are two avenues of approach, by the vagina and by the rectum, and three ways of utilizing these avenues. First, with one hand ; second, with two hands employed bimanually, either by vagina or rectum; and third, the trimanual method, by vagina and by rectum.

First.—The examination with one hand is unsatisfactory, and the ovary cannot even be felt unless abnormally displaced downward into the recto-uterine pouch.

Second —The success of the bimanual examination depends upon the downward pressure with the external hand displacing the abdominal walls in the direction of the ovary to be palpated, and thus affording a resistant plane against which the ovary can be felt by the internal hand. The internal hand must be used to invaginate the perineum, which is thus displaced upward into the pelvis. This invagination gives the examining finger, even though it be a short one, the necessary length. One, often even two inches are thus gained to the palpating finger. Care must be taken in making the pressure necessary to produce this invagination, not to stiffen all the muscles of the forearm, thus impairing the tactile sense.

The rectum is, of all others, the best avenue for approaching the structures lateral to the uterus, affording, as it does, a wide open channel throughout the whole length of the pelvis.

Where the structures cannot be reached at once through the rectum, they are brought within easy touch by bringing the uterus and ovaries into an *artificial retroposed* anteflexion, the mechanism of which was carefully described by diagrams.

Dr. Kelly had, in this way, palpated fibroid tumors on the posterior surface of the uterus, near the fundus, not as large as a pea.

Third.--The trimanual examination is conducted either by the vagina or by the rectum and vagina, assisted with the hand above. The peculiarity of this method is an *artificial desensus uteri*. The uterus is grasped with a pair of bullet forceps and drawn downward until the cervix is seen at the vaginal outlet, and while an assistant holds it in this position, the gynecologist uses his hands bimanually. To obviate the employment of an assistant, Dr. Kelly has invented an instrument, which he calls the corrugated tenaculum, flattened and roughened so that it can be readily held between the last phalanges of the third and fourth fingers and the ball of the thumb, while the index finger of the same hand, assisted by the abdominal hand above, is engaged in making a vaginal or rectal examination.

By one or the other of these methods, the uterus, broad ligaments and ovaries and tubes are within reach of a most thorough and searching examination, revealing at once the smallest abnormalities.

Dr. Wm. P. Chunod: When speaking of what should be found or what can be found at an examination, it is necessary to consider the circumstances under which the examination is made. Office examinations are the most usual and all the facilities are not usually at our command, and this circumstance should be specified and taken into account. Certain advantages in methods give certain advantages in results. Of course, where the woman has no ovaries, or where the ovaries are not in the pelvic cavity, they cannot be palpated.

Dr. Hunter Robb: I thoroughly agree with Dr. Kelley that the normal ovary can always be palpated under an anæsthetic, and also that in a large number of patients the ovary can be outlined without anæsthesia. Four years ago Dr. Kelly taught me the method of examining the ovary by invaginating the perineum, and I can testify to its utility. This lengthens out the examiner's finger and thus enables the practitioner who has a short finger to accomplish it with almost the same facility as a longer one. The corrugated tenaculum devised by Dr. Kelly may be used to advantage with patients to define the uterus and its appendages still further. No one, of course, would think of using it in inflammatory condition of the pelvic cavity.

Dr. B. B. Browne said that he had listened with much pleasure to Dr. Kelly's paper, and congratulated him upon the admirable manner in which he had systematized these valuable methods of pelvic examination—methods which most of us had been using in our gynecological practice for several years; he generally preferred the use of two fingers in the vagina as he could thus make a more satisfactory examination of the tubes and ovaries than with one finger; in many cases a more accurate idea of the adhesions can be had by getting the finger above the ovary and fixing it between the ovary and the spinal column; pulling down the uterus aids diagnosis very much.

Dr. Opie said that there were few objections to Dr. Kelly's paper, but it seemed that the elbow on the hip was incompatible with delicacy of touch; the law, as approved by Martin, being—"The more lightly the parts are touched the easier the goal is reached, and the less the force that is employed the more distinctly things are felt;" he thinks it a cruel sort of thing to drag an organ out of its position, and would like to hear how much displacement can be made with the tenaculum without producing dangerous trouble, for example, cellulitis, metritis and injury to the peri-uterine tissue; he had met a number of cases in which he had not been able to make out the ovaries; Dr. A. Martin says that he can palpate normal tubes, but Dr. Opie has never been able to reach that degree of perfection.

Dr. Neale referred to the possibility of tracing out the uterus through the anterior vaginal wall, as had been demonstrated to him by Dr. Kelly at the Hopkins Hospital clinic; he had no doubt that in a large majority of cases the normal ovary could be displaced out of its normal position and palpated or touched with ease through the vaginal walls; he believed that a great deal of difficulty in an ordinary gynecological examination was due to the fact of neglecting to empty the bladder or to employ the rectal touch.

Dr. H. P. C. Wilson said there was a large number of women in whom he was sure he could not palpate the ovaries, and he was doubtful if any one could do so. The uterus is often found fixed in the pelvis as in a mass of putty and no definite outlines can be made out; in other cases the abdominal walls are from two to four inches thick with fat and in such cases he had failed to find the ovaries.

Dr. J. Whitridge Williams said that he could certainly feel the ovaries in four cases out of five, and that he had succeeded occasionally in finding the ureter.

Dr. Moseley : The old teaching is that the ovaries cannot be palpated in their normal position. When an ovary can be found by an ordinary examination, its location may fairly be considered as abnormal. If Dr. Kelly's idea, that all men who cannot make out normal ovaries should be thrown out of the specialty, should be enforced, a large number of experienced and thoroughly informed specialists would be excluded from practice. It is practically impossible to examine every patient thoroughly enough to make out the normal ovaries in office examinations. In dispensary, and more especially in hospital practice, the case is very different.

Dr. Browne thinks that the cases in which the ovaries cannot be felt are the abnormal cases; if the symptoms point to an examination of the ovaries, they can be made out, but if necessary an anæsthetic should be given.

Dr. Kelly, in closing the discussion, said that he examines every case coming to him, vulva to ovaries, making a special note of every important organ.

When the patient complains of persistent pelvic pain, the examination is never considered complete or the diagnosis sure, without a special note as to the condition of the ovaries. I have been asked about examining the ureters by palpation. They can be felt in almost all cases, being distinctly traced from the anterior part of the pelvis back to the side of the uterus. Pressing upon a diseased ureter causes a desire to pass water, often irresistible. I prove that this structure is a ureter by catheterizing it. The catheter can be felt through the vaginal wall outside the bladder, in the ureter, and the urine collected as it comes down from the kidney drop by drop. The Fallopian tube can often but not always be made out.

"The amount of displacement of the uterus, which can be made without injury, is considerable. In normal cases it can easily, without harm or pain, be brought down to the vaginal outlet. When there is fixation, gentle traction can be made until pain is felt. In these cases I use traction with the corrugated tenaculum, and then pushing up the fundus with the finger, practice massage, stretching the adhesions. I am sure that the downward traction to the vulva, without pain, never does any harm.

Dr. J. Whitridge Williams' remarks upon the pathological specimens submitted to him by Dr. Moseley, Dr. Wilson and Dr. Opie:

"The specimens submitted by Dr. Moseley are of considerable interest, and consist of the uterine appendages from both sides. The specimen from the left side consists of the Fallopian tube, ovary and part of the broad ligament. The tube was completely occluded at its fimbriated end, but otherwise presenting nothing abnormal, except numerous small adhesions. It contained a very small amount of dirty yellow fluid, consisting of columnar ciliated epithelial cells and numerous disintegrated cells. The ovary was considerably torn and covered by very dense adhesions, while the broad ligament presented nothing of note. The specimen from the right side was an irregular mass of tissue about 5x4x1½ cm., consisting of the tube and ovary imbedded in dense adhesions. At first glance the mass appeared to be composed of two parts, a larger solid anterior portion covered by dense adhesions, and posterior to it a cystic structure about 4x1½ cm. in size. This had a bluish color, thin wall and was intimately connected with the rest of the mass. Imbedded in adhesions a piece of the ampullar end of the tube was found, which could be traced for about four cm. and then lost itself in the mass and appeared to have no connection with the above-mentioned cystic portion. The main portion of the mass on sections was shown to be composed of ovarian tissue, which was covered and completely hidden from view by very dense adhesions. It contained two tolerably fresh corpora lutea about one and a half cm. in diameter. The larger of these corpora lutea communicated by a small opening with the cystic portion above mentioned, which contained a thin reddish watery fluid containing blood cells. On cutting open this cystic portion, its walls were found perfectly smooth, with several smaller cysts projecting into it. These varied in size up to two c. in diameter and were filled with a clear watery fluid and arose directly from the ovarian tissue. On examining the scrapings from the walls of these cysts, I found that they were lined by a layer of almost flat cutoidal cells which were distinctly ciliated. These cysts could not have originated in the tear, as was readily demonstrated by their arrangement in relation to the larger cyst, and by the lining epithelium which was totally different from that of the tube. Their smooth interior precluded the idea of a ciliated papillary cystoma ; and the only probable thing for them to be were dropsical graafian follicles which had been prevented from rupturing by the dense adhesions covering them, and so attained their large size. The fact that they were lined by ciliated epithelium is not at all opposed to this supposition ; for cilia have previously been found in the dropsical graafian follicle, as was shown by Von Velits, of Budapest, about a year ago, and as I found, altogether independent of him, last spring. But as yet I have not made a sufficient number of observations to assert that all dropsical follicles are lined by ciliated epithelium. The blood in the large cyst in all probability came from the corpus luteum with which it was connected. The adhesions about the ovary were particularly dense and resisting. The diagnosis from the specimen is pelvic peritonitis, with adhesions binding down the adnexia on both sides, particularly the right side, with several very large dropsical graafian follicles.

" The specimen submitted to me by Dr. H. P. C. Wilson was a small myoma, about three cm. in diameter, and bore on one surface a piece of vaginal mucous membrane the size of a two cent piece. The tumor was submitted to me to decide whether its origin was from the anterior fornix or from the uterus itself. Sections made through the tumor and the vaginal mucous membrane readily showed it to be a myoma which was separated from the submucous tissue and epithelium by numerous bands of non-striated muscular tissue. From the presence of muscular fibre between the tumor and epithelium, I think we are justified in concluding that it was not of vaginal origin. Were it of vaginal origin it should arise from the submucous tissue and be immediately adjacent to the epithelium and not separated from it, as it was in this case, by muscular tissue. Force is lent to this conclusion by the act that vaginal fibroids are very rare indeed, and many of the reported cases, especially fibroids from the anterior fornix, had their origin in the anterior wall of the uterus, instead of the vagina.

The specimen submitted by Dr. Opie was a greatly hypertrophied posterior lip of the cervix, which measured five cm. in length and two cm. at its broadest part. Microscopically, it was found to consist of almost normal cervical tissue, with only a very slight increase of the connective tissue. Except at its cut surface, the entire mass was covered with the usual stratified epithelium.

Generally speaking, we may distinguish two forms of hypertrophy of the portio-vaginalis—follicular and diffuse or simple hypertrophy. The first form is due to an increase in number and size of the cervical glands, with frequent retention of their contents, and is quite frequent, but never attains a very great size, and is readily determined by its nodular appearance. The diffuse or simple form of hypertrophy is far more important. In this there is a general increase in all the elements that compose the cervix, though there may be a slight increase in the amount of connective tissue, as there was in this case."

WILLIAM S. GARDNER, M. D., Secretary.

